# Identification of a novel monocytic phenotype in Classic Hodgkin Lymphoma tumor microenvironment

**DOI:** 10.1371/journal.pone.0224621

**Published:** 2019-11-12

**Authors:** Ginell R. Post, Youzhong Yuan, Emily R. Holthoff, Charles M. Quick, Steven R. Post

**Affiliations:** Department of Pathology, University of Arkansas for Medical Sciences, Little Rock, Arkansas, United States of America; Virginia Commonwealth University, UNITED STATES

## Abstract

Classic Hodgkin lymphoma (CHL) characteristically shows few malignant cells in a microenvironment comprised of mixed inflammatory cells. Although CHL is associated with a high cure rate, recent studies have associated poor prognosis with absolute monocyte count in peripheral blood and increased monocyte/macrophages in involved lymph nodes. Thus, the role of monocytic infiltration and macrophage differentiation in the tumor microenvironment of CHL may be more relevant than absolute macrophage numbers to defining prognosis in CHL patients and potentially have therapeutic implications. Most studies identify tumor-associated macrophages (TAMs) using markers (e.g., CD68) expressed by macrophages and other mononuclear phagocytes, such as monocytes. In contrast, Class A Scavenger Receptor (SR-A/CD204) is expressed by tissue macrophages but not monocytic precursors. In this study, we examined SR-A expression in CHL (n = 43), and compared its expression with that of other macrophage markers. We confirmed a high prevalence of mononuclear cells that stained with CD68, CD163, and CD14 in CHL lymph nodes. However, SR-A protein expression determined by immunohistochemistry was limited to macrophages localized in sclerotic bands characteristic of nodular sclerosis CHL. In contrast, SR-A protein was readily detectable in lymph nodes with metastatic tumor, extra-nodal CHL, T cell/histiocyte-rich large B cell lymphoma, and resident macrophages in non-malignant tissues, including spleen, lymph node, liver and lung. The results of SR-A protein expression paralleled the expression of SR-A mRNA determined by quantitative RT-PCR. These data provide evidence that tumor-infiltrating monocyte/macrophages in CHL have a unique phenotype that likely depends on the microenvironment of nodal CHL.

## Introduction

Tumor-associated macrophages (TAMs) modulate the development and progression of various cancers. In classic Hodgkin lymphomas (CHL), the number of TAMs exceeds the number of malignant Hodgkin and Reed Sternberg (HRS) cells [1, 2]. The importance of TAMs in CHL is exemplified by several reports indicating that primary treatment failure and decreased overall survival of adult CHL patients is associated with TAM density, measured by CD68, CD163 or colony-stimulating factor 1 receptor (CSF1R) immunoreactivity in diagnostic lymph node specimens [3–8]. In contrast, other studies have shown no prognostic association with CD68 or CD163 expression in adult CHL [9–12]. These discrepant results may relate to diverse patient populations, disease characteristics (e.g., morphologic subtype and EBV status), the methods and threshold values used to quantify macrophage infiltration, or the differentiation/activation state of TAMs [6, 7, 13].

Antibodies commonly used to immunohistochemically define TAMs in CHL include CD68, CD163, CD14, or CSF1R. Monoclonal antibodies to CD68 (KP1 and PG-M1) recognize macrosialin, a glycoprotein associated with lysosomes/endosomes, and bind to tissue macrophages, monocytes, granulocytic precursors, and fibroblasts [14–16]. CD163 is a glycoprotein belonging to the scavenger receptor superfamily [17, 18]. CD163 immunostaining is largely restricted to peripheral blood monocytes and subpopulations of tissue macrophages [18, 19]. Similarly, CSF1R is a receptor tyrosine kinase expressed on myeloid cells, including monocytes, macrophages, and osteoclasts [20, 21]. CD14 is marker of mature monocytes, and is variably expressed on tissue macrophages and follicular dendritic cells [22, 23]. Thus, each of these commonly used “macrophage-specific” markers may be detected on monocytes and other cell types.

The class A scavenger receptor (SR-A; CD204), a pattern recognition cell surface receptor that mediates endocytosis, adhesion, phagocytosis, and cell signaling, is induced during monocyte to macrophage differentiation [24–28]. SR-A expression is restricted to tissue macrophages with little detectable expression in other cell types [29, 30]. SR-A expression has been detected on TAMs in gliomas [31], pancreatic [32], kidney [33], esophageal [34], lung cancer [35], and T cell lymphoma [36]. SR-A expression by TAMs is associated with tumor progression in some, but not all of these tumor types [37]. Based on cell expression pattern, SR-A may be a more specific marker that distinguishes TAMs from other cell types and monocytes.

Because SR-A is a marker of differentiated macrophages, we compared the expression pattern of CD68, CD163, CD14 and SR-A in CHL tumor microenvironment. We found that most of the cells identified with CD68 and/or CD163 were negative for SR-A, with SR-A staining limited to macrophages in the collagenous bands characteristic of nodular sclerosis CHL (NSCHL). Notably, SR-A expression was readily detected in resident macrophages of the spleen, lymph node, liver, and lung, and in both nodal T cell/histiocyte-rich large B cell lymphoma (THRLBCL) and nodal metastatic carcinoma. The results of SR-A protein expression by immunohistochemistry paralleled SR-A mRNA expression determined by quantitative RT-PCR. The lack of SR-A expression on infiltrating cells in the microenvironment of nodal CHL, independent of histologic subtype, Epstein Barr viral (EBV) status and relapsed disease, indicates that these cells are immunophenotypically distinct from resident macrophage populations and TAMs in THRLBCL and nodal metastatic carcinoma.

## Materials and methods

### Sample selection

In this retrospective case analysis, we reviewed 43 cases of CHL (41 nodal and 2 non-nodal-liver and spleen), 4 cases of THRLBCL, and 5 lymph nodes with metastatic colon cancer. The diagnosis of CHL and THRLBCL were reviewed and confirmed by two clinical hematopathologists (GP and CY) based on morphologic features and immunohistochemical studies as defined by the WHO Classification [38, 39]. Nonmalignant tissues known to have differentiated macrophages, including spleen (n = 2), lymph node (n = 1), liver (n = 2), and lung (n = 2) were selected as positive controls for antibody staining. Formalin-fixed and paraffin-embedded tissue (FFPE) specimens were sectioned at 5 μm for immunohistochemical staining or at 10 μm for RNA isolation. This study was reviewed by the University of Arkansas for Medical Sciences Institutional Review Board (IRB) which determined that this project is not human subject research as defined in 45 CFR 46.102.

### Immunohistochemistry

All cases of CHL were immunostained with antibodies to SR-A and CD68 with a subset also immunostained with antibodies to CD163 (n = 18) and/or CD14 (n = 22). Cases of THRLBCL, metastatic tumor, and control tissues were also immunostained with antibodies to SR-A, CD68, CD163, and CD14. Immunohistochemistry was performed using two monoclonal antibodies to SR-A (MRS1; clone OTI9ES; 1:150 and clone UMAB 246; 1:600; OriGene), CD68 (Clone KP-1; Roche), CD163 (Clone MRQ-26 Cellmarque), CD14 (Clone EPR3653; Ventana), CD30 (Clone Ber-H2; Ventana) and CD20 (Clone L26 Ventana). Antigen retrieval and immunostaining for SR-A was performed manually in the UAMS Experimental Pathology Core using heat-induced epitope retrieval in 1mM EDTA in 10mM Tris buffer (pH 9.0) at >15 PSI (Biocare) for 20 minutes. Tissue sections were then incubated with goat anti-mouse (SR-A) followed by avidin-biotin-peroxidase complex (Dako) and developed with diaminobenzadine. All other antibodies were processed on an automated stainer (Ventana).

The intensity, pattern, and percent of CD68, CD163, CD14 and SR-A (clone OTI9ES) positive cells for each case was recorded by a third pathologist blinded to the final pathology (MQ). A second SR-A antibody (clone UMAB246) was used to stain a representative case for each tissue in order to confirm staining results with OTI9ES, but was not quantified. Statistical analysis was performed using a one-way ANOVA and Tukey’s post-hoc test to compare relative staining in control tissues, lymph nodes with metastatic tumor and CHL.

### RNA isolation and reverse transcription

Total RNA was extracted from sections of FFPE tissues using an RNA isolation kit (Qiagen RNeasy® FFPE; Valencia CA) according to the manufacturer’s recommendations. Briefly, two 10 μm thick sections from paraffin embedded tissue blocks were deparaffinized, incubated in lysis buffer containing proteinase K and DNase, and then applied to RNA-binding columns. The columns were washed with ethanol and RNA eluted in water. RNA samples were analyzed spectrophotometrically on a Nanodrop (NanoDrop 1000 Spectrophotometer, Thermo Fisher Scientific Inc, Wilmington, DE). Only samples with OD 260/280 ratio 1.7–2.1 were used for reverse transcription. cDNA was synthesized from 1 μg RNA by using the high capacity cDNA reverse transcription kit (Applied Biosystems) according to manufacturer’s recommended protocol.

### Real time quantitative PCR

A subset of recent (<4 years old) CHL lymph node cases (2 NSCHL, 2 MCCHL, 1 LRCHL), lymph nodes with metastatic tumor, and control tissues were randomly selected for quantitative real-time PCR (qRT-PCR) analysis. qRT-PCR was performed using predesigned Applied Biosystems Taqman® real-time PCR gene expression assays (ThermoFisher) using 25 ng of cDNA and 10 nM gene-specific DNA primers [SR-A (Assay ID# Hs00234007), CD163 (Assay ID# Hs00174705), CD68 (Assay ID# Hs02836816), and GAPDH (#Hs03929097)] in a 20 μl reaction. An Applied Biosystems StepOne® Plus Real-Time PCR System was used for amplification using the assay-specified optimized parameters. Total RNA that was not reverse transcribed was used as a negative control. SR-A and CD163 expression in tissues were normalized to CD68 in the same sample, and then the normalized expression was compared with expression in THP-1 cells (ATCC, TIB202) that were differentiated to macrophages by culturing for 3 days in medium containing 200 ng/ml phorbol myristic acid (PMA).

## Results

The forty-three cases of CHL identified in the pathology database and analyzed in this study are summarized in **Table 1**. Of the 43 cases, 33 (77%) were subclassified as nodular sclerosis (NSCHL) variant, six cases (14%) were mixed cellularity (MCCHL), two cases were lymphocyte rich (LRCHL) (4.5%), and two cases (4.5%) were extra-nodal (i.e., unclassified). The majority of cases involved lymph nodes (38/43), were EBV negative (19 of 27 cases with known EBV status), and represented diagnostic tumor specimens (33/43); the remaining ten specimens were relapsed disease.

**Table 1:** Summary of cases.

	Number (% total)	Site (n)	EBV+ (n = 27)	Relapsed (n = 10)
**Nodal CHL Subtype**				
Nodular Sclerosis	33 (77%)	Lymph node (31) Mediastinum (2)	3 1	8 0
Mixed Cellularity	6 (14%)	Lymph node (5) Mediastinum (1)	1 1	1 0
Lymphocyte Rich	2 (4.5%)	Lymph node (2)	0	0
**Extra-nodal** (unclassified)	2 (4.5%)	Spleen (1) Liver (1)	1 1	1 0

### SR-A expression in nodal CHL

**Fig 1:**
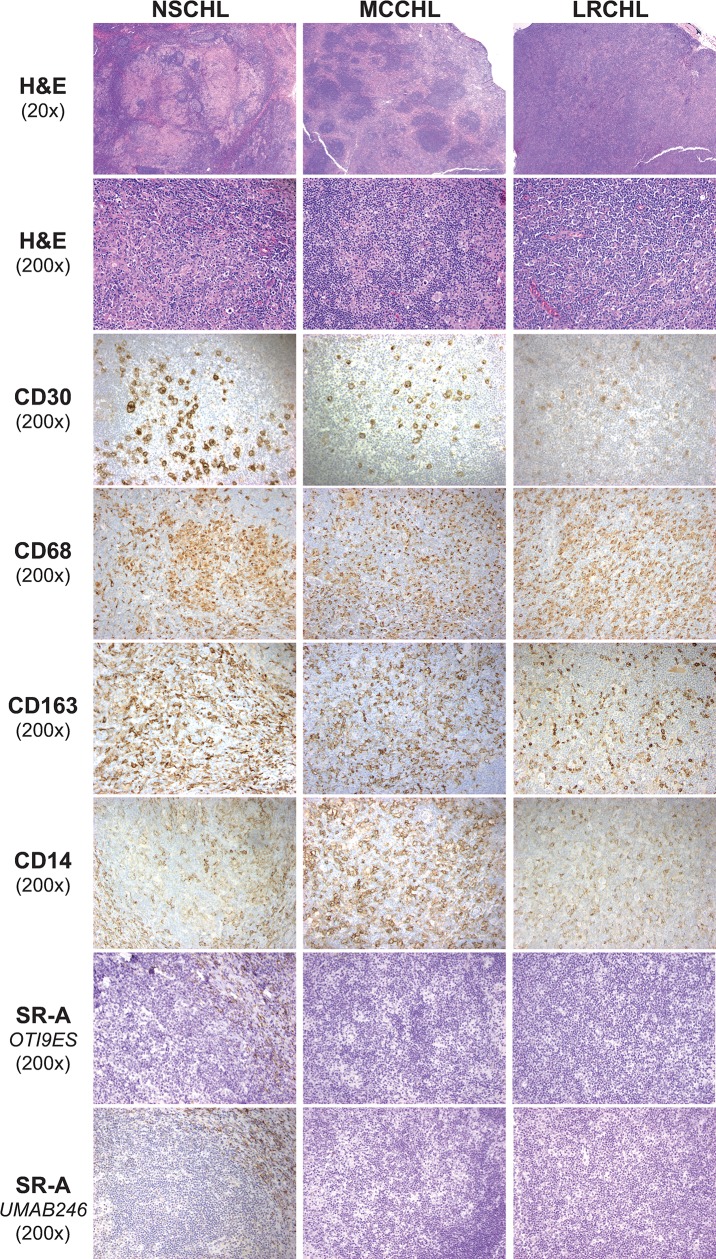
Expression pattern of monocyte/macrophage markers in nodal CHL. Shown are hematoxylin and eosin (H&E) and immunostaining patterns of different cellular markers in FFPE sections from representative CHL morphologic variants [nodular sclerosis (NSCHL, left panels), mixed cellularity (MCCHL, middle panels) and lymphocyte rich (LRCHL, right panels)]. CD30 immunoreactivity was used to identify malignant HRS cells. Antibodies to CD68, CD163 and CD14 were used to stain monocyte/macrophages, and staining patterns for these antibodies compared with that of SR-A using two different monoclonal antibodies (clones OTI9ES and UMAB246). Notably, monocyte/macrophages in proximity to the malignant cells in nodal CHL were positive for these three monocyte/macrophage markers. In contrast, antibody staining for SR-A in nodal CHL was limited to the fibrotic bands characteristic of NSCHL.

CHL histologic variants (NSCHL, MCCHL, LRCHL) were immunostained for CD30 to identify malignant HRS cells, and for CD68, CD163, and CD14 to identify TAMs (**Fig 1**). Consistent with previous reports, CD68, CD163, and CD14 antibodies strongly stained cells in the CHL tumor microenvironment in each of the variants. A majority of cells that were positive for CD68 (reference marker for macrophages) were also stained with CD163 and CD14. In contrast, the pattern of SR-A staining was distinctly different than that of CD68, CD163, and CD14. Specifically, SR-A immunostaining was negative in macrophages that were in close proximity to CD30 positive HRS tumor cells (i.e., TAMs). When detected, SR-A positive macrophages were only associated with the sclerotic bands associated with NSCHL. The staining pattern of SR-A in nodal CHL was similar when detected using a different monoclonal SR-A antibody (clone UMAB246).

**Fig 2:**
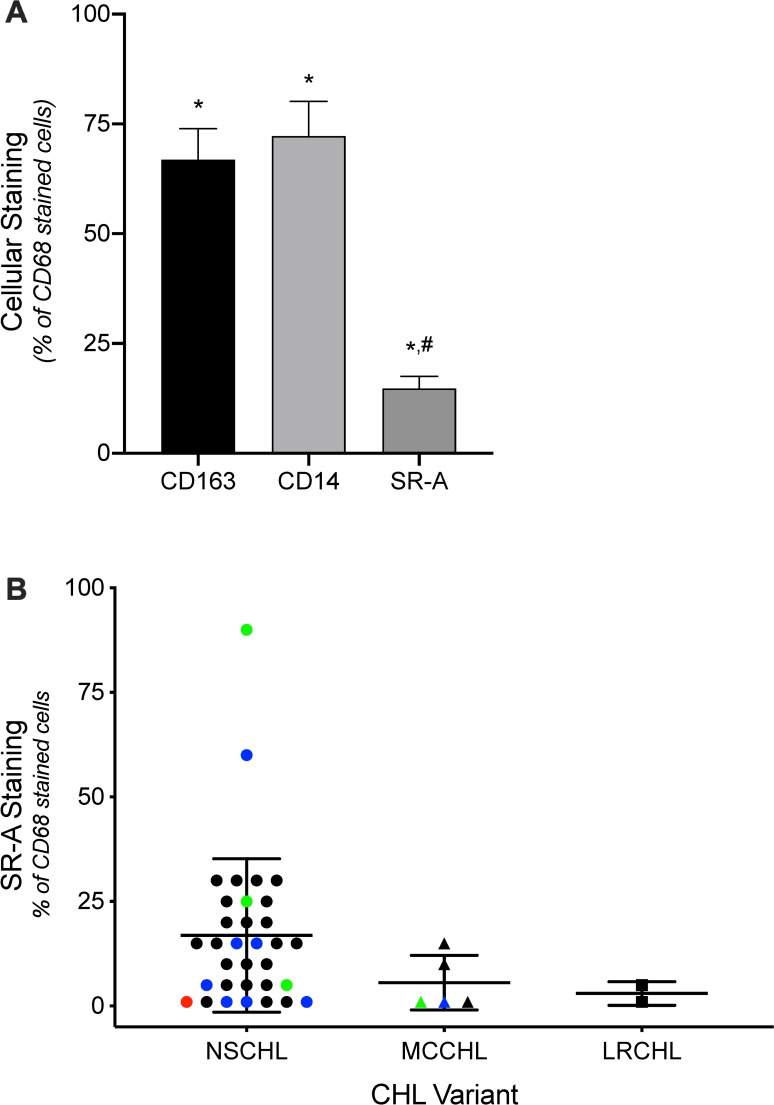
Analysis of CD163, CD14, and SR-A expression compared to CD68 in CHL lymph nodes. Tissue sections of nodal CHL stained with CD68, CD163, CD14, and SR-A (OTI9ES) were scored by a pathologist. (A) The graph depicts the percent of CD68 positive cells that were also stained with CD163, CD14, or SR-A. * denotes significant differences (p<0.05) from CD68 assessed by one-way t-test; # denotes significant difference (p<0.05) from CD163 and CD14 determined by one-way ANOVA and Tukey’s post-hoc test. (B) SR-A staining relative to CD68 in nodal CHL based on morphologic subtype. As shown in Fig 1, SR-A staining of the NSCHL was detected in fibrotic bands (Green symbol: EBV positive cases; Blue symbol: relapsed disease; Red symbol: EBV positive, relapsed disease).

The overall results comparing CD163, CD14, and SR-A (OTI9ES) staining of TAMs in CHL with that of CD68 (reference marker for macrophages) are summarized in **Fig 2**. There was strong overlap (67%, p = 0.0003) between CD163 and CD68 staining of cells in tissues (Fig 2A). Similarly, CD14 stained 72% (p = 0.002) of the CD68 positive cells. There was no difference (p = 0.81) between CD163 and CD14 with respect to staining CD68 positive cells. Importantly, SR-A showed a pattern of staining in CHL that was significantly different than CD68, CD163, and CD14, with an overall staining of 15% (p<0.0001) of the CD68 positive cells (Fig 2A). Consistent with SR-A staining in fibrotic bands of NSCHL, this subtype tended to have more SR-A positive cells than other CHL subtypes, although this difference was not significant (p>0.05); Fig 2B). Although the number of cases was low, there was no apparent differences in staining overlap based on positive EBV status (green), relapsed disease (blue) or both (red). However, given that SR-A was not detected in the tumor microenvironment of any of the nodal CHL cases, it is reasonable to conclude that the lack of SR-A expression in this environment is independent of EBV status and relapsed disease.

### SR-A expression in tissue macrophages

**Fig 3:**
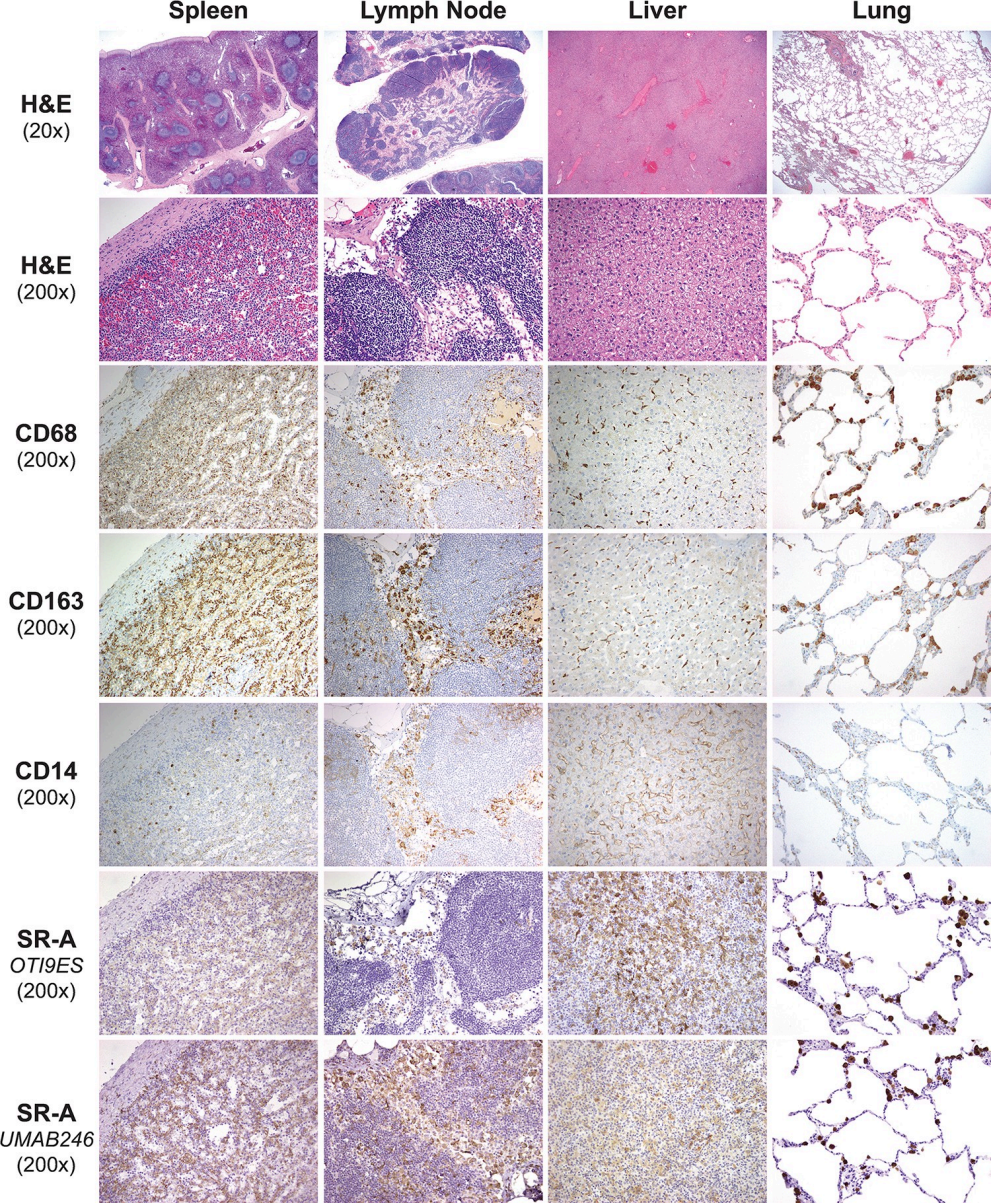
Expression pattern of monocyte/macrophage markers in spleen, lymph node, liver, and lung. Shown are representative H&E and immunostaining patterns of resident tissue monocyte/macrophages in FFPE sections of spleen, lymph node, liver, and lung. SR-A staining (OTI9ES and UMAB246) shows a similar pattern to CD68 and CD163 in the splenic cords and sinus histiocytes (lymph nodes), Kuppfer cells (liver) and alveolar macrophages (lung). CD14 staining was different from the other macrophages markers in splenic red pulp cords, Kuppfer cells (liver), and alveolar macrophages (lung).

Resident macrophages are found in several tissues including splenic red pulp cords, lymph node, liver (Kupffer cells), and lung (alveolar macrophages). Given the unexpected absence of SR-A staining in TAMs compared to CD68, CD163 and CD14 in nodal CHL, we examined the staining pattern of these immunohistochemical markers in tissue-resident macrophage populations. Both CD68 and CD163 stained red pulp mononuclear cells in the spleen, sinus histiocytes in the lymph node, hepatic Kupffer cells, and alveolar macrophages of the lung (**Fig 3**). The staining pattern of CD14 differed from that of CD68 and CD163, being positive in a small subset of mononuclear cells in the splenic red pulp, nodal and hepatic sinuses, pulmonary parenchyma and hepatic sinusoidal lining cells; but negative in alveolar macrophages and hepatic Kupffer cells. As reported previously [23], CD14 also uniquely labeled follicular dendritic cells in the germinal centers of lymph nodes. In contrast to the staining pattern of nodal CHL, SR-A staining of tissue-resident macrophages in these tissues was similar to both CD68 and CD163 (**Fig 3**).

**Fig 4:**
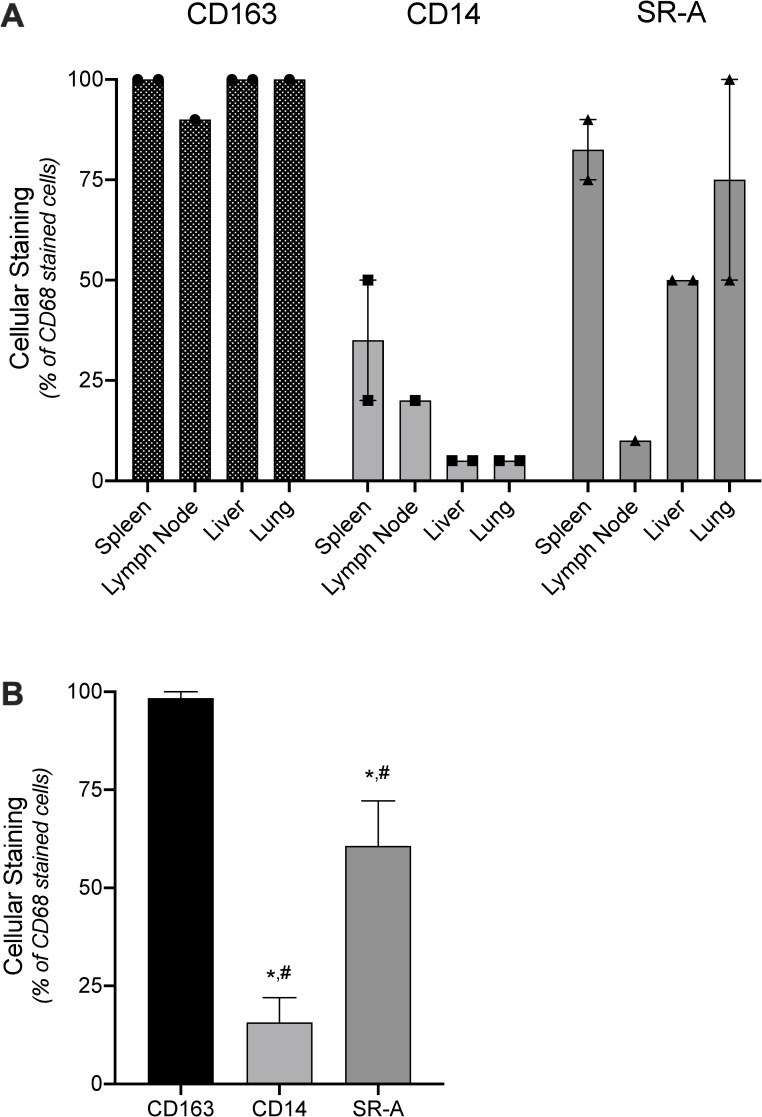
Analysis of CD163, CD14 and SR-A expression compared to CD68 in resident tissue monocyte/macrophages. A) Tissue-resident monocyte/macrophages in FFPE sections of spleen, lymph node, liver, and lung were immunostained and scored for the percent of CD68 positive cells that also stained with CD163, CD14, or SR-A (OTI9ES). Symbols represent the percent of CD68 positive cells that were co-stained in individual samples. Bars depict the means and range for the individual tissue type. B) Average of CD68 positive monocyte/macrophages that also stained with CD163, CD14, or SR-A across tissue types. Bars depict the mean and SEM. * denotes significant differences (p<0.05) from CD68 assessed by one-way t-test; # denotes significant difference (p<0.05) from CD163 determined by one-way ANOVA and Tukey’s post-hoc test.

The overall results comparing CD163, CD14, and SR-A staining of resident mononuclear cells with that of CD68 are summarized in **Fig 4**. There was no difference between CD163 and CD68 staining of cells in tissues (p = 0.363); whereas, CD14 stained 16% (p<0.0001) of the CD68 positive cells. The low degree of overlap reflects CD14 staining many cells that were not CD68 positive (Fig 3). Although the overall cellular staining of SR-A in tissues was lower than that of CD68 (60%; p = 0.014), SR-A showed a staining pattern similar to both CD68 and CD163 (Fig 3), with the lowest amount seen in lymph nodes where SR-A staining was limited to mononuclear cells in the sinus (Fig 3).

### SR-A expression in other malignancies and non-nodal CHL

**Fig 5:**
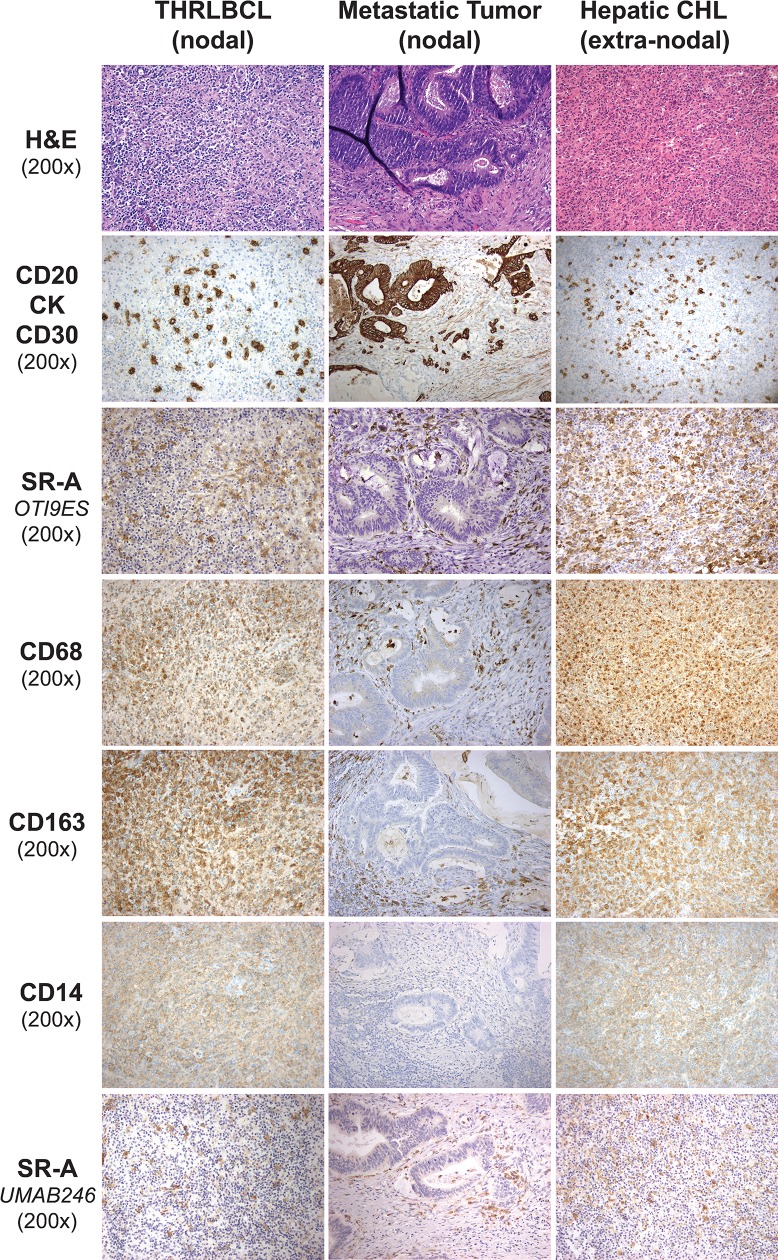
Expression pattern of monocyte/macrophage markers in nodal T cell/histiocyte-rich large B cell Lymphoma (THRLBCL), nodal metastatic colon cancer, and hepatic CHL. Shown are representative H&E and immunostaining patterns of monocyte/macrophages in FFPE sections of nodal THRLBCL (left panels), nodal metastatic colon carcinoma (center panels) and extra-nodal CHL (right panels). CD20 identifies malignant B cells in THRLBCL, cytokeratin (CK) shows malignant metastatic tumor cells, and CD30 detects malignant HRS cells in CHL. SR-A staining (OTI9ES and UMAB246) shows a similar pattern to CD68, CD163 and CD14 in each tissue.

**Fig 6:**
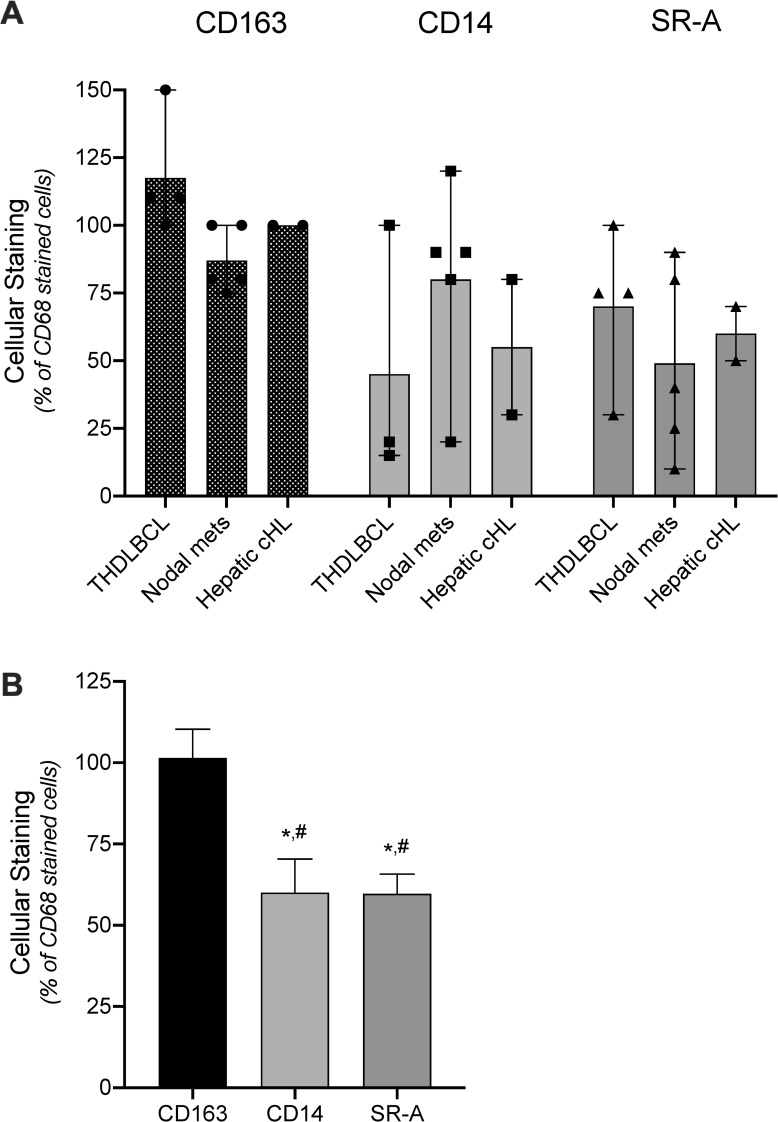
Analysis of CD163, CD14, and SR-A expression in TAMs. A) Tumor-associated macrophages present in sections of THRLBCL, metastatic colon cancer, and hepatic CHL were immunostained and scored for the percent of CD68 positive cells that also stained with CD163, CD14, or SR-A (OTI9ES). Symbols represent the percent of CD68 positive cells that were co-stained in individual samples. Bars depict the means and range for the individual tumor types. B) Average of CD68 positive TAMs that also stained with CD163, CD14, or SR-A across tumor types. Bars depict the mean and SEM. * denotes significant differences (p<0.05) from CD68 assessed by one-way t-test; # denotes significant difference (p<0.05) from CD163 determined by one-way ANOVA and Tukey’s post-hoc test.

Having established that SR-A staining differed between nodal CHL and tissue-resident macrophages, we examined the staining patterns of the monocyte/macrophage markers in other tumor microenvironments including lymph nodes with metastatic colon cancer, CHL involving the liver, and THRLBCL. Similar to CHL, THRLBCL is a B cell lymphoma with a small number of malignant B cells in a background of reactive T cells and histiocytes. The goal of these studies was to determine if SR-A stains TAMs in lymph nodes involved by a different B cell lymphoma and metastatic carcinoma and in CHL involving non-nodal tissue. As shown in **Fig 5**, CD68, CD163, CD14, and SR-A staining (OTI9ES and UMAB246) were detected in nodal THRLBCL and lymph nodes with metastatic colon cancer. Interestingly, in contrast to SR-A staining of nodal CHL, SR-A expression in TAMs of extra-nodal CHL (liver) was comparable to both CD68 and CD163 (**Fig 5**). Using CD68 as a reference macrophage marker, we found the overall cellular overlap in these malignancies was 101% (p = 0.88) for CD163, 60% (p = 0.06) for CD14, and 60% (p = 0.02) for SR-A (**Fig 6**).

### SR-A expression by qRT-PCR

**Fig 7:**
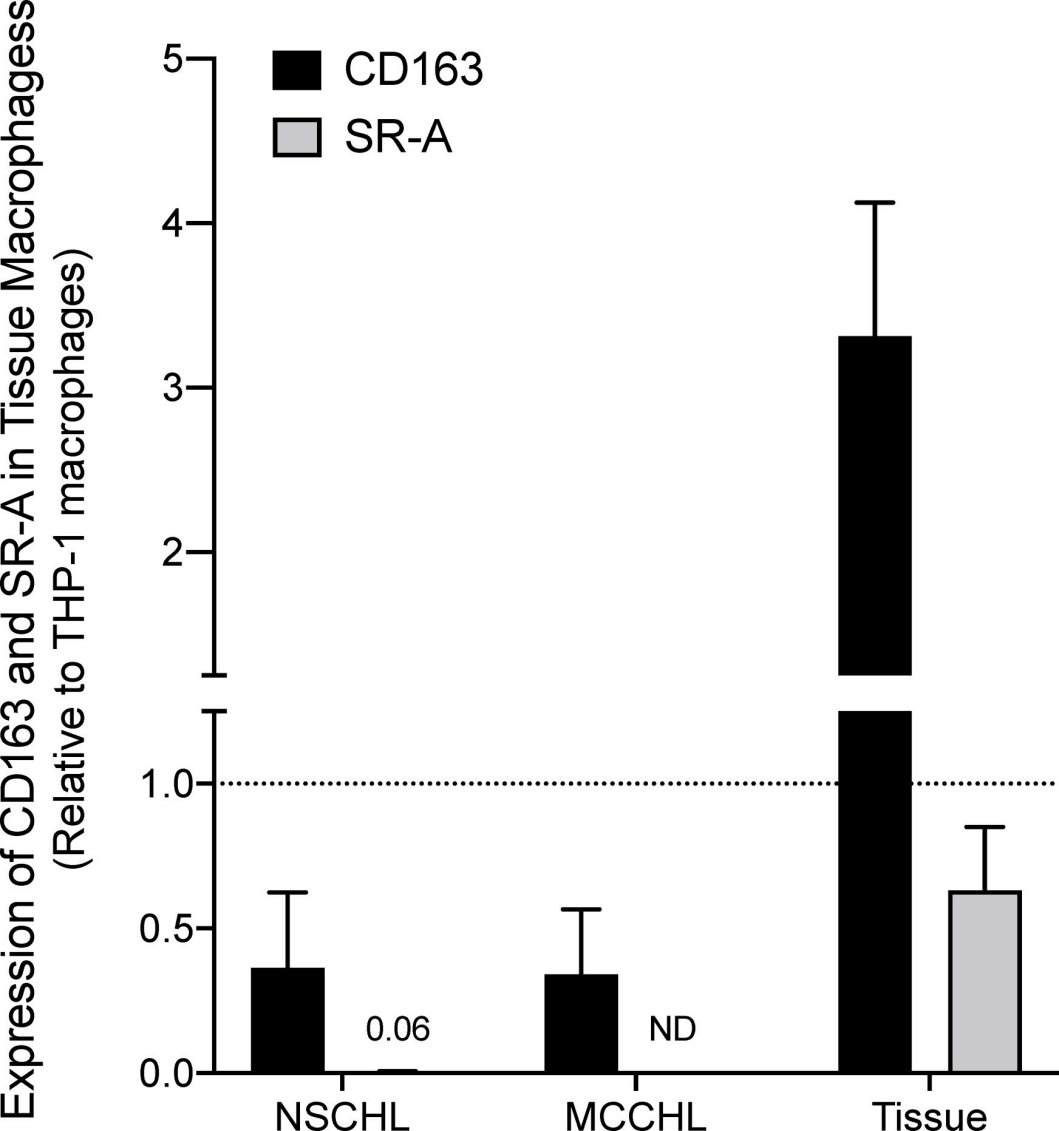
CD163, CD14 and SR-A expression in nodal CHL, lung and liver assessed by qRT-PCR. Total RNA was prepared from FFPE sections of nodal NSCHL (n = 2) and MCCHL (n = 2), from tissue (lung and liver), and differentiated THP-1 macrophages (positive control). After reverse transcription, qRT-PCR was used to quantify expression of CD68, CD163, and SR-A. Relative expression of CD163 and SR-A in macrophages was calculated by normalizing their Ct values to that of CD68 in the same sample (ΔCt), and then to their expression in THP-1 macrophages (ΔΔCt). CD163 expression was readily detected in nodal CHL and lung/liver (tissue), and to a lesser extent in NSCHL and MCCHL. SR-A expression was readily detected in tissue, but was only detected at very low levels in NSCHL and not detected (ND) in MCCHL.

The absence of SR-A staining determined by immunohistochemistry indicates that TAMs in nodal CHL are phenotypically different than resident tissue macrophages, and TAMs in other malignancies including extranodal CHL, THRLBCL, and lymph nodes with metastatic carcinoma. To provide additional support for this observation, we quantified CD68, CD163, and SR-A expression by qRT-PCR of RNA isolated from FFPE blocks in a subset of cases and differentiated human THP-1 macrophages (**Fig 7**). To account for potential differences in macrophage numbers, the expression of CD163 and SR-A were normalized to CD68 in the same sample, and then results expressed as a fraction of CD68 expression detected in THP-1 macrophages. CD163 expression was readily detected in nodal CHL (NSCHL, MCCHL variants), lung and liver (tissue) and did not differ between NSCHL and MCCHL subtypes (**Fig 7A**). In contrast, SR-A expression, which was readily detected in both lung and liver, could not be reliably detected in nodal CHL, except at low levels in NSCHL (**Fig 7B**).

## Discussion

Increased macrophage infiltration has been associated with adverse prognosis in adult nodal CHL. This association was originally established from results of gene expression profiling that showed over-representation in the gene signatures of monocyte/macrophages and from immunohistochemistry using antibodies to CD68 [4]. To provide additional insight regarding the immunophenotype of TAMs in CHL, cases of nodal CHL were immunostained for SR-A. In contrast to CD68, CD163, and CD14 which are expressed by monocytes and macrophages, SR-A expression is restricted to differentiated tissue macrophages. Our results show that cellular SR-A immunoreactivity is similar to that of CD68 in nonmalignant tissue-resident macrophages (Figs 3 and 4), lymph nodes with metastatic carcinoma, nodal THRLBCL and extra-nodal CHL (Figs 5 and 6). In contrast, the mononuclear cells in nodal CHL strongly express CD68, CD163 and CD14, but SR-A expression in nodal CHL was not detected in the cell-rich tumor microenvironment and was limited to the sclerotic bands characteristic of the NSCHL (Fig 1). The findings of decreased SR-A protein expression by immunohistochemistry paralleled qRT-PCR results showing a lack of SR-A mRNA in nodal MCCHL and low-level transcripts in nodal NSCHL. SR-A staining of resident tissue macrophages, macrophages in extra-nodal CHL, and macrophages other tumor microenvironments support the conclusion that the lack of SR-A staining of mononuclear cells in nodal CHL reflects a monocytic phenotype that is unique to the microenvironment of nodal CHL.

Notably, SR-A expression was identified on CD68 positive macrophages in the fibrotic bands characteristic of NSCHL (Fig 1). Malignant HRS cells induce IL-6 secretion by fibroblasts [40], and secrete cytokines such as IL-13, TNF-α and TGF-β that not only modulate SR-A expression in human monocyte-derived macrophages [41–44], but also increase fibroblast proliferation and deposition of a collagen-rich extracellular matrix characteristic of the NSCHL [45]. SR-A mediates the adhesion of macrophages to extracellular matrix and may enhance their retention at sites of matrix deposition (e.g., sclerotic bands) [46–56]. This matrix-rich environment may be distinct from that of the cell-rich, matrix-poor environment in nodal CHL. Expression of SR-A only in the sclerotic bands characteristic of nodal NSCHL suggests that macrophages in this matrix-rich microenvironment may be exposed to factors, such as collagen, that regulate macrophage retention and monocyte-macrophage differentiation.

The absence of SR-A expression in nodal CHL could arise from different mechanisms. First, SR-A expression may be selectively down-regulated in macrophages in the immediate tumor microenvironment. Macrophage migration inhibitory factor (MIF) and macrophage colony-stimulating factor (M-CSF), cytokines that regulate SR-A expression [57], are present in the CHL tumor microenvironment. Treatment of primary monocytes with M-CSF stimulates their differentiation into macrophages and increases SR-A expression [21, 58–61]. Other factors, including TLR ligands [62], high glucose [63], and ligands for SR-A [59] also increase macrophage expression of SR-A. Interestingly, following removal of stimulus, SR-A expression returns to its original basal level [59]. In contrast, IL-6, IFN-γ, TNF-α, and TGF-β reportedly reduce SR-A expression by 50–70% in human monocyte-derived macrophages [41–44]. However, to our knowledge there are no reports of signals that completely inhibit macrophage expression of SR-A. Therefore, it seems unlikely that the absence of SR-A expression by TAMs in the immediate tumor microenvironment in nodal CHL results from the selective repression of SR-A transcription.

SR-A expression is absent in monocytes and becomes expressed during their differentiation into macrophages [24–28]. Thus, another mechanism for the lack of SR-A expression in TAMs of nodal CHL is that SR-A expression was not upregulated during the differentiation of recruited monocytes. In support of such a possibility, GM-CSF reportedly significantly reduces, although does not completely block, the induction of SR-A expression during the differentiation of human monocyte-derived macrophages [64]. Although we cannot exclude the possibility that failure to detect SR-A in nodal CHL results from a failure to induce SR-A during macrophage differentiation, it seems unlikely because TAMs in extra-nodal CHL, TAMs in other tumors, and resident tissue macrophages all express SR-A indicating that such a mechanism would be unique to TAMs in the tumor microenvironment of nodal CHL.

An alternative explanation is that TAMs in the microenvironment of nodal CHL are not fully differentiated macrophages. In addition to not expressing SR-A, this possibility is suggested by the finding that the inflammatory cells strongly express the monocyte marker CD14, as well as CD163 and CD68, antigens shared by monocytes and macrophages. This is relevant because adverse outcome in CHL patients is predicted by an absolute monocyte count >750 cells/μL in peripheral blood at diagnosis [65]. Further, in CHL patients with relapsed disease post-allotransplant, tumor-infiltrating mononuclear cells are derived from circulating monocytes and not resident macrophages [66]. Functionally, Vari et al recently showed that in adult CHL PD-L1/CD163/CD14 expressing mononuclear cells suppress NK cell activation [67]. Together, these results indicate a potential role for recruited monocytes in CHL pathogenesis.

Overall, our results indicate that in nodal CHL, regardless of morphologic variant, EBV status or relapsed disease, monocytes are recruited into the tumor microenvironment but do not differentiate into a TAM phenotype characteristic of tissue-resident macrophages or TAMs in other settings. Therefore, identifying the unique factors in the nodal microenvironment of CHL that underlie monocyte infiltration and result in restricted differentiation will advance our understanding of the complexity of TAM phenotypes and may suggest novel therapeutic strategies for treating CHL patients.

## Supporting information

S1 File Supporting data. (XLSX)
